# The Efficacy and Safety of the mTOR Signaling Pathway Activator, MHY1485, for *in vitro* Activation of Human Ovarian Tissue

**DOI:** 10.3389/fgene.2020.603683

**Published:** 2021-02-04

**Authors:** Shuang Wu, Yining Wang, Haixiang Ding, Ningxia Sun, Yan Liu, Liang Wang, Fei Sheng, Honghong Zhang, Fu Yang, Wen Li

**Affiliations:** ^1^Shanghai Key Laboratory of Embryo Original Diseases, International Peace Maternity and Child Health Hospital, School of Medicine, Shanghai Jiao Tong University, Shanghai, China; ^2^Department of Reproductive Medicine Center, Changzheng Hospital, Second Military Medical University, Shanghai, China; ^3^Department of Medical Genetics, Second Military Medical University, Shanghai, China

**Keywords:** premature ovarian insufficiency, mTOR signaling pathway, MYH1485, *in vitro* activation, safety, DNA methylation

## Abstract

**Background:**

Premature ovarian insufficiency (POI) is characterized by abnormal ovarian function before the age of 40. POI showed that primordial follicles developed in disorder. mTOR signaling plays a vital role in the process of follicle development. It has been verified that the mTOR signaling pathway activator, MHY1485, can promote primordial follicle development in mice. We considered that MHY1485 would be a promising fertility preservation method for POI patients.

**Methods:**

The fragmented ovarian tissues of normal woman was cultured with activator MHY1485 *in vitro*, and then the control and activated ovaries were transplanted into the kidney capsules of ovariectomized mice. We then used the Infinium Human Methylation EPIC BeadChip to verify the DNA methylation level of ovarian tissues, thus exploring the effectiveness of them.

**Results:**

MHY1485 stimulated mTOR, S6K1, and rpS6 phosphorylation. Cultured with MHY1485, ovarian weights increased and endocrine function was restored. The number of growing follicles was increased. The *in vitro* activation process did not induce histological changes or abnormal DNA methylation occurrence.

**Conclusion:**

MHY1485 for *in vitro* activation (IVA) is effective for ovarian rejuvenation and is a potential therapeutic treatment for POI patients.

## Introduction

Premature ovarian insufficiency (POI) is a clinical syndrome in women (age <40 years old), characterized by menstrual disturbance with an elevated FSH level and low estradiol content. Most POI patients develop follicular dysfunction or the premature exhaustion of the follicle pool (De Vos et al., 2010; Webber et al., 2016). The pregnancy rate of POI patients is only about 1–5% (van Kasteren and Schoemaker, 1999; Bidet et al., 2011), even with conventional assisted reproductive technology (ART). Some POI patients of childbearing age still have a difficult time conceiving a baby. *In vitro* activation (IVA) of ovarian tissue is a new assisted reproductive therapy method for women. This study aims to meet POI patients’ fertility needs by using IVA of ovarian tissue. Kawamura et al. (2013) have reported that Hippo signaling disruption by ovarian tissue fragmentation combined with AKT stimulation of ovarian follicles contributed to the first successful pregnancy in POI patients. Additionally, mTOR pathway activators could both activate primordial follicles and stimulate secondary follicle growth (Adhikari et al., 2010; Ren et al., 2015; Sun et al., 2015).

The Mammalian Target of Rapamycin (mTOR) pathway is a downstream signaling pathway that can be regulated by the AKT, and was widely studied in the field of cancer for issues such as tumorigenesis, metastasis, and therapy (Saxton and Sabatini, 2017; Mossmann et al., 2018). The mTOR signaling pathway activator MHY1485 would increase the phosphorylation of downstream ribosomal S6 kinase (S6K1) and ribosomal protein S6 (rpS6) in the ovarian tissue of mice. Culturing ovaries with MHY1485 promoted follicle development and MHY1485-treated mice could deliver healthy pups (Cheng et al., 2015). Therefore, we speculated that MHY1485 might be an effective activator for the IVA of human ovarian tissue. IVA is expected to be a promising infertility treatment strategy that enables POI patients to conceive their own genetic children. Unfortunately, the magnitude of risk for IVA is still poorly understood, so an evaluation of its safety is truly necessary.

DNA methylation regulates gene expression in many ways and is of great significance for gametogenesis and early embryogenesis (De Munck et al., 2015). DNA methylation abnormalities can affect the growth of offspring, leading to genetic diseases (Greenberg and Bourc’his, 2019). Also, ART can increase the risk of gene-related diseases. Follicles are vulnerable during the time window where they are abnormally sensitive to external factors (El Hajj and Haaf, 2013). Professor Qiao et al. and De Munck et al. have found that DNA methylation alters the oocytes in different situations (Yan et al., 2014; De Munck et al., 2015), although whether the culture and activation of ovarian tissue will affect global DNA methylation patterns in ovarian tissues has not yet been determined.

Our study aimed to establish a standardized mouse model of a human ovarian tissue activation-xenograft and then use this model to verify the efficacy and safety of the mTOR signaling pathway activator MHY1485 for human ovarian primordial follicle activation.

## Materials and Methods

### Materials *in vitro* Culture and Activation

The ovary tissue of patients (*n* = 11) of non-ovarian factors containing primordial follicles were collected using excision surgery at the Department of Obstetrics and Gynecology in Shanghai Changzheng Hospital. All the participants are transsexual patients that had normal ovarian function and were not under hormonal treatment before the surgery. This study is approved by the Institutional Medical and Ethical Review Committee of Second Military Medical University. Clinical characteristics of cases were shown in Table 1. After being cut into 1 mm^3^ cubes, cortical tissues were placed on culture plate inserts (Millipore) and cultured in 400 μl of DMEM/F12 (Gibco) containing 10% human serum albumin (CSL Behring), 1% penicillin-streptomycin solution (HyClone), 0.3 IU/ml FSH, and 0.05 mg/ml L-ascorbic acid under a membrane insert to cover ovaries with a thin layer of medium. Ovaries were treated with 1–20 μM MHY1485 (10 mM in 1 ml DMSO, APExBIO, United States) and cultured for 3 h before immunoblotting analysis. Control ovaries were treated with solvent only. Other pairs of ovaries were cultured for 2 days with a medium change after 24 h of culture. Ovaries were fixed with formalin for histological analysis or prepared for transplantation before weighing.

**TABLE 1:** Clinical characteristics of transsexual patients.

Patients, *n* = 11	Mean ± SEM
Age, years	30.543.50
Menstrual cycles, days	28.181.66
BMI	21.323.03
**Endocrinology**	
FSH, mIU/ml	4.991.40
Estradiol, pg/ml	195.04157.78
LH, mIU/ml	10.5514.70

### Immunoblotting Analysis

Pairs of ovarian tissue were cultured for 3 h, and proteins were extracted using RIPA buffer containing a protease inhibitor cocktail (APExBIO, United States) and phosphatase inhibitors (Roche, Switzerland). Protein concentrations were determined by BCA protein assay (Beyotime Institute of Biotechnology, China). Equal amounts of protein lysates were separated on 12% PAGE Bis-Tris gels (Epizyme) and transferred onto nitrocellulose membranes. Proteins were visualized, and images were obtained using an Odyssey infrared imager.

### Ovarian Transplantation

Pairs of fragmented ovarian tissue (with or without MHY1485 treatment) from the same donor were cultured for 2 days *in vitro* with a medium change every 24 h. Then, the host animals were anesthetized and fragments were randomly transplanted under separate sides of the kidney capsule in the same ovariectomized adult (6–8-week-old) SCID mice. Three days after transplantation, hosts received daily i.p. injections of FSH (1 IU/day) for 28 days to promote follicle development. Thirty days after transplantation, grafts from the same recipient were recovered and weighed before histological analyses. Furthermore, we transplanted the activated fragments into ovariectomized adult SCID mice so that we could dynamically monitor the hormone levels.

### Histological Analysis

Two days after IVA and 1 month after transplantation, some ovaries were fixed with formalin, paraffin-embedded, and then cut into continuous 8-μm-thick sections. One fragment was collected for calculation of the number of follicles and the experiment was repeated three times. Sections were stained with hematoxylin and eosin (HE) reagent for further observation. Then we selected one slide of every five and each sample was chosen 15 pieces in the lump to calculate total follicles and the percentage of different phases of follicles to total follicles, thus avoiding counting the same slide twice or missing a follicle.

### Enzyme-Linked Immunosorbent Assay (ELISA)

Hormone levels in mice were monitored dynamically before and after ovariectomy and ovary transplantation by ELISA. Venous blood samples were collected from the eye socket veins in mice. Serum was extracted at three time points: before castration, after castration, and 30 days after transplantation. Serum was separated and stored at −80°C until use. Repeated freeze/thaw cycles were avoided. Serum FSH and AMH levels were measured by using an ELISA kit for mice following the manufacturer’s instructions (Westang, Shanghai, China).

### Genome-Wide DNA Methylation Profiling

Genomic DNA (gDNA) was extracted from ovarian tissues using a QIAamp DNA Mini Kit (Qiagen, Hilden, Germany) according to the manufacturer’s protocol. Approximately 1 μg of high-quality gDNA was bisulfite-converted using the EZ DNA methylation kit (Zymo Research, Cambridge Bioscience, Cambridge, United Kingdom) according to the manufacturer’s instructions. Genome-wide DNA methylation profiles were generated with an Infinium Methylation EPIC BeadChip Array (Illumina, San Diego, CA, United States). Bisulfite-converted DNA was used for the whole-genome amplification reaction, enzymatic fragmentation, precipitation, and resuspension in hybridization buffer. Subsequent steps were carried out according to the standard Infinium HD Assay Methylation Protocol Guide. The BeadChip images were captured using the Illumina iScan system (Illumina, San Diego, CA, United States).

### Statistical Analysis

The results are presented as the mean ± SEM of three or more independent assays. Statistical significance was analyzed using Student’s *t* test, one-way analysis of variance (ANOVA), the Shapiro–Wilk *W* test, or Bartlett’s test for equal variances. DNA methylation data were analyzed with Bayesian modeling. *P*-values < 0.05 were considered to be statistically significant.

## Results

### MHY1485 Activate the mTOR Signaling Pathway in Human Ovary

**FIGURE 1:**
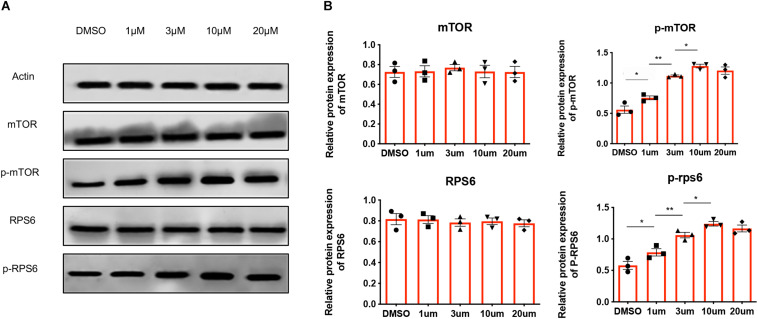
MHY1485 treatment can stimulate the phosphorylation of proteins in the mTOR pathway. **(A)** Treatment of 1 μM, 3 μM, and 10 μM MHY1485 led to a dose-dependent increase in the phosphorylation of mTOR as well as downstream RSP6 protein levels without affecting total mTOR rpS6 expression. Treatment of 20 μM did not increase those protein levels further in comparison to 10 μM MYH1485. **(B)** Experiments were repeated three times. All data were compared with that of the control group. Data was applied to non-parametric tests for statistical analysis and presented as the mean ± SEM *P < 0.05, **P < 0.01, and the differences are significant.

Given that MHY1485 could activate the mTOR pathway in mouse ovaries and lead to the birth of offspring mice, we investigated the activation of the mTOR signaling pathway in human ovarian tissues treated with MHY1485. Human ovarian cortices were fragmented and incubated for 3 h with different concentrations of MHY1485 (1 μM, 3 μM, 10 μM, and 20 μM) before immunoblotting analyses. As shown in Figures 1A,B, treated with MHY1485, the phosphorylation levels of downstream mTOR and RPS6 protein were augmented with the additive amount, however, the total protein level of mTOR and RPS6 were not altered. It is worth noticing that there is no significant difference between the 10 μM MYH1485 group and 20 μM MYH1485 group, which indicates that 10 μM may be the maximal activation concentration of MYH1485 to the mTOR pathway. Therefore, these results demonstrated that MHY1485 could effectively activate the mTOR signaling pathway in the human ovary.

### MHY1485 Effectively Promote Human Follicle Growth and Restore Endocrine Functions

**FIGURE 2:**
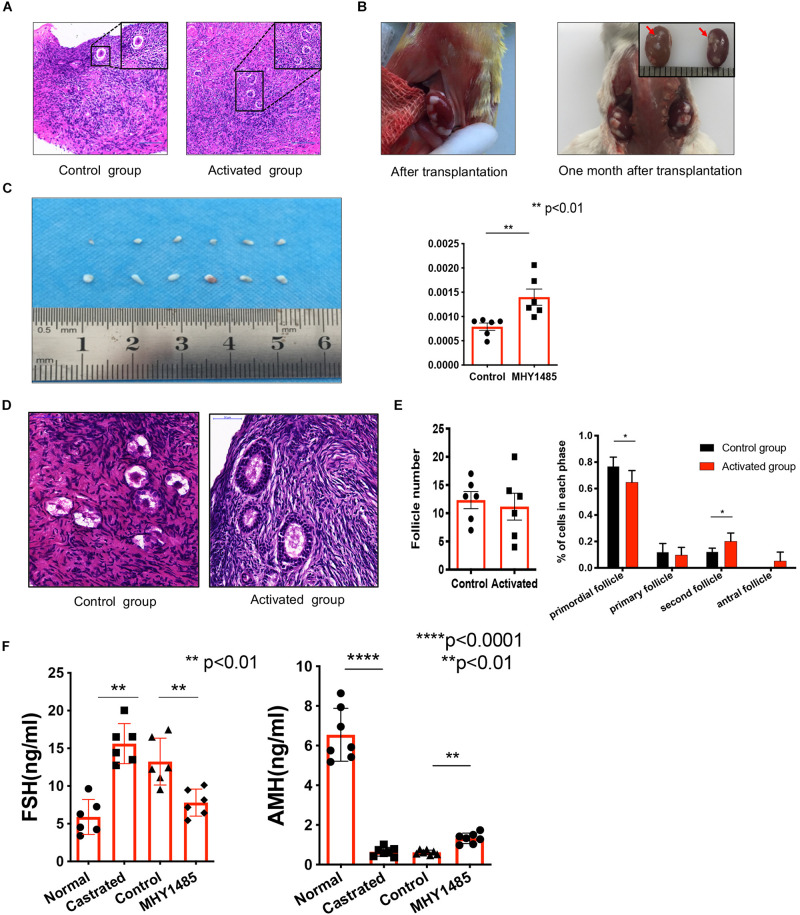
Treatment of MHY1485 can effectively promote human follicle growth and restore endocrine functions. Human ovarian fragments were cultured with 10 μM MHY1485-added media (activated group) or culture media only (control group) *in vitro*. Histological analyses showed that there was no obvious necrosis or morphological change when the two groups were compared. **(A)** Control group treated without MYH1485, Bars: 100 μm. Activated group treated with MYH1485, Bars: 100 μm. **(B)** No abnormal histological affected on human ovarian tissues after transplantation. **(C)** Graft weight changed. After treatment of MHY1485 one month, grafts weight increased compared with the control group. Data was applied paired *t* test for statistical analysis and presented as the mean ± SEM **P < 0.01, *N* = 6. **(D)** Following histological analyses, the development of larger growing follicles was observed in the MYH1485-treated ovarian tissues, Bars: 50 μm. Whereas primary follicles remained dormant in the untreated ovarian tissues, Bars: 50 μm. **(E)** Follicle counting results (**right:** total follicle numbers per fragment; **left:** follicle dynamics), showed that the number of growing follicles increased after activation with 1 month. Data was applied paired *t* test for statistical analysis and presented as the mean ± SEM *P < 0.05, *N* = 6. **(F)** FSH (ng/ml) elevated after the surgical castration of the SCID mice and decreased 1 month after activated grafts were transplanted into host mice, **P < 0.01, *N* = 6. AMH (ng/ml) dropped after surgical castration and slightly increased 1 month after activation (normal:untreatment; castrated:after castrated without other treatment; control:castrated with normal medium treatment; MHY1485: castrated with MHY1485 medium treatment). Data was applied ANOVA for statistical analysis and presented as the mean ± SEM **P < 0.01, ****P < 0.0001, *N* = 6.

Next, we examined whether MYH1485 could lead to histological abnormalities of human ovarian tissues *in vitro* and *in vivo*. Firstly, we treated fragmented human ovarian tissues with 10 μM MHY1485-added culture media (activated group) or culture media only (control group) for 2 days *in vitro*. The subsequent histological results showed that there was no obvious necrosis or morphological change in both the control group and activated group (Figure 2A). *In vivo*, fragmented ovaries from the above activated and control groups were then transplanted under the bilateral kidney capsule in the same ovariectomized adult (6–8-week-old) severe combined immune-deficiency (SCID) mice, which received daily intraperitoneal (i.p.) injections of follicle stimulating hormone (FSH) (1 IU/day) to promote follicle development. One month after transplantation, we observed that all the ovarian tissue survived in the mice and there was no obvious tissue necrosis (Figure 2B). Therefore, MHY1485 treatment showed no abnormal histological effects on human ovarian tissues.

Then, we explored the effects of MYH1485 on the growth of human follicles. We recovered and weighed the paired grafts (activated and control groups) again 1 month after transplantation. Before transplantation, the mean value of activated group and control group achieved very similar performances (*P* > 0.05, *N* = 6). While after treatment of MHY1485, as shown in Figure 2C, the graft’s weight significantly increased compared with the control group (^∗∗^*P* < 0.01). The histological analyses indicated that the development of larger growing follicles (Figure 2D) was apparent in the MYH1485-treated ovarian tissue, while the primary follicles (Figure 2D) remained dormant in the untreated ovarian tissue. Moreover, follicles counting results showed that the treatment of MYH1485 effectively increased the number of growing follicles (Figure 2E). Furthermore, we examined the serum levels of anti-Mullerian hormone (AMH) and FSH in the ovariectomized adult SCID mice before and after being transplanted with MHY1485-activated human ovarian tissues by ELISA assays. The results showed that ovariectomy remarkably increased the FSH level and decreased the AMH level (Figure 2F[[AQ19]]), however, the FSH level (Figure 2F) significantly decreased and the AMH level slightly elevated after the transplantation. Together, treatment of MHY1485 could effectively promote human follicle growth and restore endocrine functions.

### The Culture and Activation Process of Ovarian Tissue *in vitro* Did Not Cause Abnormal DNA Methylation

**FIGURE 3:**
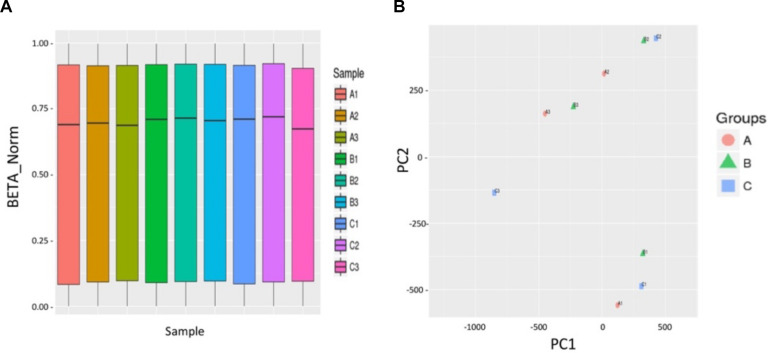
The culture and activation process of ovarian tissue *in vitro* did not cause abnormal DNA methylation. **(A)** No significant differences in total genome DNA methylation between three groups of human ovarian tissues were observed: normal group A (A1, A2, A3) (normal human ovarian fragment), control group B (B1, B2, B3) (treated with culture media only), and activated group C (C1, C2, C3) (treated with 10 μM activator MHY1485). The abscissa indicates the nine sample names, and the ordinate represents the methylation level (standardized beta value). **(B)** The PCA chart indicates the similarity between the samples by the method of decreasing dimensions. The three groups did not cluster together, indicating that the similarity of the samples within the same group was poor, and there were no significant differences between the three groups. | Delta Beta| ≥ 0.12 (*P* < 0.01) is used to screen out the different sites, Delta Beta ≥ 0.12 indicates that the degree of methylation is increased, and Delta Beta ≤ –0.12 indicates that the degree of methylation is decreased. Three volcano plots between groups show the differential of the methylation sites.

We further used a human high-throughput DNA methylation microarray (Infinium Human Methylation EPIC BeadChip: 850k chip) to analyze the effects of the activator MHY1485 on the methylation status of the human genome in the ovary. We designed the three following groups: the normal group (normal human ovarian fragment), control group (treated with culture media only), and activated group [treated with activator (10 μM MHY1485)]. The samples of group A, B, and C were derived from the same patient. We randomly selected one tissue of three patients, respectively for repeated experiments. We used | Delta Beta| ≥ 0.12 (*P* < 0.01) as the cutoff value to indicate a difference; Delta Beta ≥ 0.12 indicated that the degree of methylation was increased, and Delta Beta ≤ −0.12 indicated that the degree of methylation was decreased. The results showed that there were no significant differences in the whole-genome DNA methylation patterns of the three groups of human ovarian tissues (Figure 3A). As shown in Figure 3B, we measured the similarity between the samples by decreasing dimensions and found that the three groups could not be clustered together, indicating that there were no significant differences among the three groups.

## Discussion

A very small number of etiologies for POI can be clearly diagnosed in POI patients, and the treatment for them is not always effective. Earlier studies revealed that IVA technology could be a new infertility treatment strategy for POI patients to conceive their own genetic children (Kawamura et al., 2013; Zhai et al., 2016).

Recent studies have demonstrated that the mTOR activator MHY1485 is able to promote primordial follicle growth in mice. However, its efficacy and safety in humans is uncertain. Our studies have finally illustrated that the mTOR activator MYH1485 could increase the phosphorylation of mTOR downstream proteins and stimulate human follicle growth. One month after the transplantation and gonadotropin injections, we found that MYH1485 could increase the graft’s weight compared with the control treatment. Larger growing follicles were apparent in MYH1485-treated ovarian tissues, while dormant follicles were present in the untreated ovarian tissue. The above results showed that short-term exposure to an appropriate concentration of MHY1485 could activate human primary follicles.

In our study, we initially performed an ovary castration surgery on SCID mice, which was confirmed a success by the altered serum FSH level and AMH level. FSH was elevated after the ovariectomy while the AMH is declined. Compared with the control group, the activated group displayed a reverse effect of the FSH and AMH levels, manifesting the survival of human ovarian tissue in them. Adding MHY1485 to the normalized human ovarian tissue xenograft model could help to restore endocrine functions. In clinical practice, we usually use FSH and AMH to predict ovarian function in women. Increased FSH levels are commonly observed in women with various conditions, including POF, hyperpituitarism, and Turner syndrome. Serum AMH has a strong relationship with the number of antral follicles (Fanchin et al., 2003; Nardo et al., 2007; Majumder et al., 2010), and can improve cycle-to-cycle reproducibility (Aflatoonian et al., 2009). Together, the present findings suggested that short-term exposure to the mTOR signaling activator might be a promising therapeutic approach for infertile POI patients.

Several pregnancies have been reported due to the application of IVA-transplantation technology to human ovaries (Kawamura et al., 2013; Zhai et al., 2016). However, the biosafety of the drug and the possible effect on any resulting offspring have not been strongly confirmed. A system review in animals have suggested that ART can lead to adverse pregnancy outcomes, such as an increased risk of birth defects and an increased rate of low-birth-weight infants (Hansen et al., 2013). The normal maintenance of DNA methylation played an important role in oocyte and embryo development through the regulation of gene expression (El Hajj and Haaf, 2013). The application of additional ART can perturb gene regulation, which can lead to abnormal epigenetic phenotypes and genetic defects (Grace and Sinclair, 2009; Laprise, 2009; Denomme and Mann, 2012), though the vast majority of ART children appear to be healthy. A study has successfully activated neonatal mouse ovaries through short-term IVA using an inhibitor of PTEN and a PI3K activating peptide without abnormal epigenetic changes (Li et al., 2010). But long-term monitoring of the progeny mice was absent in the study. In 2012, another researcher, via long-term monitoring of the IVA-mice, found that the progeny mice were reproductively active and did not have any overt symptoms of chronic diseases (Adhikari et al., 2012). All experiments using IVA have indicated that IVA is a safe and effective method for infertility therapy. The human high-throughput DNA methylation microarray was performed to analyze the effects of MHY1485 on the methylation status of the human genome in the ovary. We have not found obvious abnormal DNA methylation changes among group A, B, and C. However, MHY1485 may affect ovaries in other ways, such as protein modification, alternative splicing, and so on, which deserves further research. Moreover, oocytes are preferred to detect DNA methylation level, however, mature oocytes are difficult to obtain. In addition, the number of oocytes is limited and not enough to complete DNA methylation experiments. Therefore, we selected ovarian tissue to analyze the effects of MHY1485 on the methylation levels. We could perform Single cell DNA methylation sequencing in future research. Furthermore, it has been demonstrated that stem cells could promote follicular development, granulosa cell proliferation, and secretion function by improving the local microenvironment of the ovary (Liu et al., 2019). Therefore, we thought that *in vitro* treatment may not affect DNA methylation and also may have a relatively small effect on the oocytes.

Our study aimed to establish a human ovarian tissue activation-xenograft animal model and verify the efficacy and safety of the mTOR signaling pathway activator MHY1485 for human ovarian primordial follicle activation. We explored, for the first time, the effect of IVA on DNA methylation in ovarian tissue. Then, we conducted a preliminary assessment of the safety of the procedure and provided experimental evidence for the IVA technique for clinical application in terms of fertility preservation in POI patients. Unfortunately, due to a lack of conclusive research, many decisions in human ART thus far have not been based on evidence. Genome-wide methylation analysis based on next-generation microarrays will hopefully better explain the effects of ART-based manipulations. Our study evaluated the safety of IVA in ovarian tissue in terms of epigenetic effects, namely DNA methylation, but there are several limitations. Patients will accept long-term superovulation and IVF-ET therapy after the transplantation of *in vitro* activated ovarian tissue. It is difficult to assess whether IVA will actually induce increased birth risks.

We will further obtain mature human eggs through long-term superovulation of IVA grafts, analyze the epigenetic effects in activated and control eggs, and then use the data to supplement the evidence for the assessment of safety. The genetic safety and epigenetic effects of IVA technology remain to be further explored.

## Data Availability Statement

The raw data supporting the conclusions of this article will be made available by the authors, without undue reservation.

## Ethics Statement

The studies involving human participants were reviewed and approved by the Institutional Medical and Ethical Review Committee of Second Military Medical University. The patients/participants provided their written informed consent to participate in this study. The animal study was reviewed and approved by the Institutional Medical and Ethical Review Committee of Second Military Medical University. Written informed consent was obtained from the individual(s) for the publication of any potentially identifiable images or data included in this article.

## Author Contributions

WL and FY conceived and designed the experiments. SW, YW, and HD performed the experiments and analyzed the data. SW and YW wrote the main manuscript. NS, LW, and YL were involved in data curation. FS prepared the table. HZ revised the manuscript. All authors reviewed the manuscript.

## Conflict of Interest

The authors declare that the research was conducted in the absence of any commercial or financial relationships that could be construed as a potential conflict of interest.

## Supplementary Material

Supplementary Figure 1 Fragments’ weight between control group and activated group before grafting is approximately the same. Data was applied to paired *t* test for statistical analysis and presented as the mean ± S *N* = 6.

## Abbreviations

POI: Premature ovarian insufficiency
ART: Assisted reproductive technology
IVA: *In vitro* activation
SCID: Severe combined immune-deficiency
FSH: Follicle stimulating hormone
AMH: anti-Mullerian hormone
HE: hematoxylin and eosin
ELISA: Enzyme-linked immunosorbent assay.

## References

[B1] Adhikari D. Gorre N. Risal S. Zhao Z. Zhang H. Shen Y. (2012). The safe use of a PTEN inhibitor for the activation of dormant mouse primordial follicles and generation of fertilizable eggs. *PLoS One* 7:e39034. 10.1371/journal.pone.0039034 22761722 PMC3384593

[B2] Adhikari D. Zheng W. Shen Y. Gorre N. Hämäläinen T. Cooney A. J. (2010). Tsc/mTORC1 signaling in oocytes governs the quiescence and activation of primordial follicles. *Hum. Mol. Genet.* 19 397–410. 10.1093/hmg/ddp483 19843540 PMC2798719

[B3] Aflatoonian A. Oskouian H. Ahmadi S. Oskouian L. (2009). Prediction of high ovarian response to controlled ovarian hyperstimulation: anti-Müllerian hormone versus small antral follicle count (2-6 mm). *J. Assist. Reprod. Genet.* 26 319–325. 10.1007/s10815-009-9319-5 19543966 PMC2729857

[B4] Bidet M. Bachelot A. Bissauge E. Golmard J. L. Gricourt S. Dulon J. (2011). Resumption of ovarian function and pregnancies in 358 patients with premature ovarian failure. *J. Clin. Endocrinol. Metab.* 96 3864–3872. 10.1210/jc.2011-1038 21994953

[B5] Cheng Y. Kim J. Li X. X. Hsueh A. J. (2015). Promotion of ovarian follicle growth following mTOR activation: synergistic effects of AKT stimulators. *PLoS One* 10:e0117769. 10.1371/journal.pone.0117769 25710488 PMC4340052

[B6] De Munck N. Petrussa L. Verheyen G. Staessen C. Vandeskelde Y. Sterckx J. (2015). Chromosomal meiotic segregation, embryonic developmental kinetics and DNA (hydroxy)methylation analysis consolidate the safety of human oocyte vitrification. *Mol. Hum. Reprod.* 21 535–544. 10.1093/molehr/gav013 25833840

[B7] De Vos M. Devroey P. Fauser B. C. J. M. (2010). Primary ovarian insufficiency. *Lancet* 376 911–921. 10.1016/S0140-6736(10)60355-8 20708256

[B8] Denomme M. M. Mann M. R. W. (2012). Genomic imprints as a model for the analysis of epigenetic stability during assisted reproductive technologies. *Reproduction* 144 393–409. 10.1530/REP-12-0237 22956517

[B9] El Hajj N. Haaf T. (2013). Epigenetic disturbances in in vitro cultured gametes and embryos: implications for human assisted reproduction. *Fertil. Steril.* 99 632–641. 10.1016/j.fertnstert.2012.12.044 23357453

[B10] Fanchin R. Schonäuer L. M. Righini C. Guibourdenche J. Frydman R. Taieb J. (2003). Serum anti-Müllerian hormone is more strongly related to ovarian follicular status than serum inhibin B, estradiol. FSH and LH on day 3. *Hum. Reprod.* 18 323–327. 10.1093/humrep/deg042 12571168

[B11] Grace K. S. Sinclair K. D. (2009). Assisted reproductive technology, epigenetics, and long-term health: a developmental time bomb still ticking. *Semin. Reprod. Med.* 27 409–416. 10.1055/s-0029-1237429 19711251

[B12] Greenberg M. V. C. Bourc’his D. (2019). The diverse roles of DNA methylation in mammalian development and disease. *Nat. Rev. Mol. Cell Biol.* 20 590–607. 10.1038/s41580-019-0159-6 31399642

[B13] Hansen M. Kurinczuk J. J. Milne E. de Klerk N. Bower C. (2013). Assisted reproductive technology and birth defects: a systematic review and meta-analysis. *Hum. Reprod. Update* 19 330–353. 10.1093/humupd/dmt006 23449641

[B14] Kawamura K. Cheng Y. Suzuki N. Deguchi M. Sato Y. Takae S. (2013). Hippo signaling disruption and Akt stimulation of ovarian follicles for infertility treatment. *Proc. Natl. Acad. Sci. U.S.A.* 110 17474–17479. 10.1073/pnas.1312830110 24082083 PMC3808580

[B15] Laprise S. L. (2009). Implications of epigenetics and genomic imprinting in assisted reproductive technologies. *Mol. Reprod. Dev.* 76 1006–1018. 10.1002/mrd.21058 19484754

[B16] Li J. Kawamura K. Cheng Y. Liu S. Klein C. Liu S. (2010). Activation of dormant ovarian follicles to generate mature eggs. *Proc. Natl. Acad. Sci. U.S.A.* 107 10280–10284. 10.1073/pnas.1001198107 20479243 PMC2890455

[B17] Liu R. Zhang X. Fan Z. Wang Y. Yao G. Wan X. (2019). Human amniotic mesenchymal stem cells improve the follicular microenvironment to recover ovarian function in premature ovarian failure mice. *Stem Cell Res. Ther.* 10:299. 10.1186/s13287-019-1315-9 31578152 PMC6775662

[B18] Majumder K. Gelbaya T. A. Laing I. Nardo L. G. (2010). The use of anti-Müllerian hormone and antral follicle count to predict the potential of oocytes and embryos. *Eur. J. Obstetr. Gynecol. Reprod. Biol.* 150 166–170. 10.1016/j.ejogrb.2010.02.029 20223579

[B19] Mossmann D. Park S. Hall M. N. (2018). mTOR signalling and cellular metabolism are mutual determinants in cancer. *Nat. Rev. Cancer* 18 744–757. 10.1038/s41568-018-0074-8 30425336

[B20] Nardo L. G. Christodoulou D. Gould D. Roberts S. A. Fitzgerald C. T. Laing I. (2007). Anti-Müllerian hormone levels and antral follicle count in women enrolled in in vitro fertilization cycles: relationship to lifestyle factors, chronological age and reproductive history. *Gynecol. Endocrinol.* 23 486–493. 10.1080/09513590701532815 17852428

[B21] Ren Y. Suzuki H. Jagarlamudi K. Golnoski K. McGuire M. Lopes R. (2015). Lhx8 regulates primordial follicle activation and postnatal folliculogenesis. *BMC Biol.* 13:39. 10.1186/s12915-015-0151-3 26076587 PMC4487509

[B22] Saxton R. A. Sabatini D. M. (2017). mTOR Signaling in Growth. *Metab. Dis. Cell* 168 960–976. 10.1016/j.cell.2017.02.004 28283069 PMC5394987

[B23] Sun X. Su Y. He Y. Zhang J. Liu W. Zhang H. (2015). New strategy for in vitro activation of primordial follicles with mTOR and PI3K stimulators. *Cell Cycle* 14 721–731. 10.1080/15384101.2014.995496 25590233 PMC4615062

[B24] van Kasteren Y. M. Schoemaker J. (1999). Premature ovarian failure: a systematic review on therapeutic interventions to restore ovarian function and achieve pregnancy. *Hum. Reprod. Update* 5 483–492. 10.1093/humupd/5.5.483 10582785

[B25] Webber L. Davies M. Anderson R. Bartlett J. Braat D. Cartwright B. (2016). ESHRE Guideline: management of women with premature ovarian insufficiency. *Hum. Reprod.* 31 926–937. 10.1093/humrep/dew027 27008889

[B26] Yan J. Zhang L. Wang T. Li R. Liu P. Yan L. (2014). Effect of vitrification at the germinal vesicle stage on the global methylation status in mouse oocytes subsequently matured in vitro. *Chi. Med. J.* 127 4019–4024. 25430442

[B27] Zhai J. Yao G. Dong F. Bu Z. Cheng Y. Sato Y. (2016). In Vitro activation of follicles and fresh tissue auto-transplantation in primary ovarian insufficiency patients. *J. Clin. Endocrinol. Metab.* 101 4405–4412. 10.1210/jc.2016-1589 27571179 PMC5095246

