# Supplementary figures and images for: The Efficacy and Safety of the mTOR Signaling Pathway Activator, MHY1485, for in vitro Activation of Human Ovarian Tissue
Source: Front Genet. 2021 Feb 4;11:603683. doi: 10.3389/fgene.2020.603683 (PMC7890121; doi:10.3389/fgene.2020.603683)

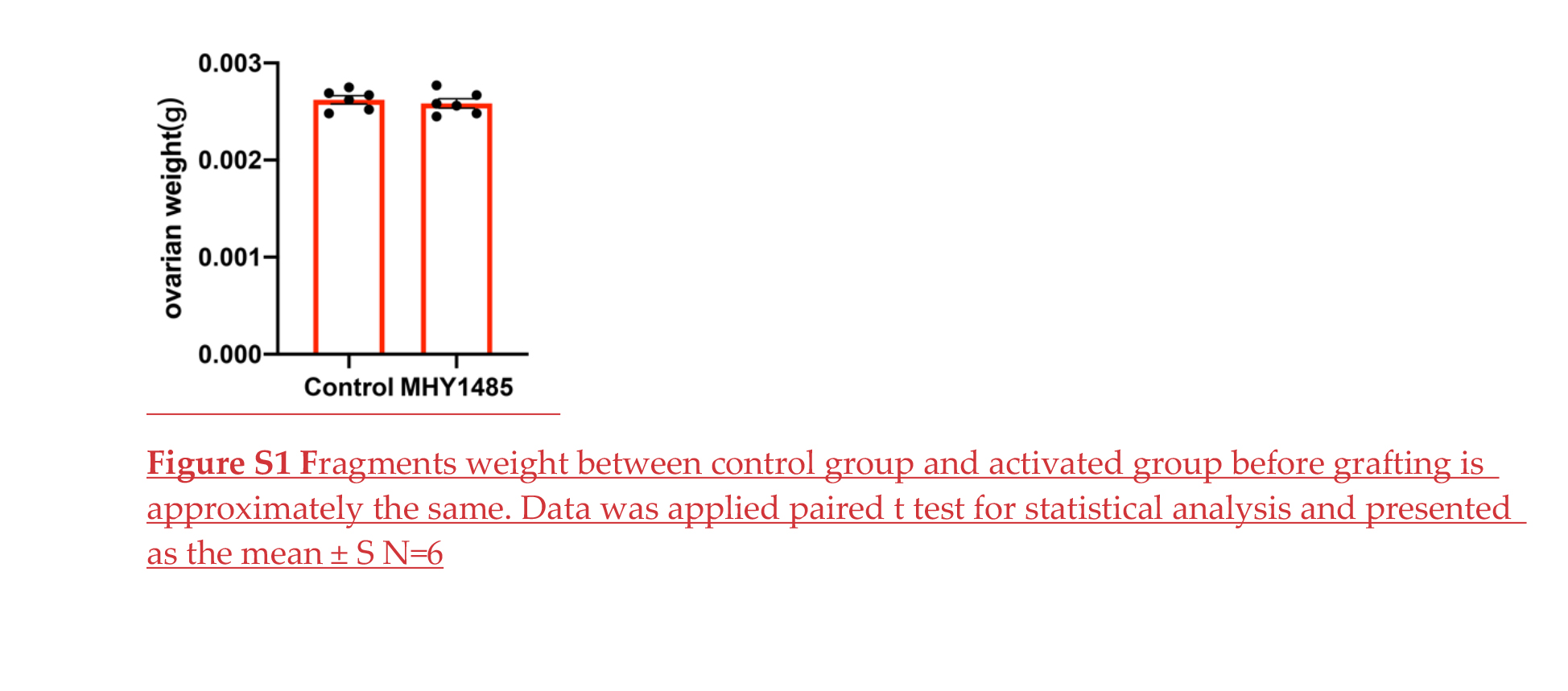

Supplement: Supplementary Figure 1 — Fragments’ weight between control group and activated group before grafting is approximately the same. Data was applied to paired t test for statistical analysis and presented as the mean ± S N = 6. [file Image_1.JPEG]
